# Integration of predictive-corrective incompressible SPH and Hodgkin-Huxley based models in the OpenWorm in silico model of C. elegans

**DOI:** 10.1186/1471-2202-14-S1-P209

**Published:** 2013-07-08

**Authors:** Michael Vella, Andrey Palyanov, Padraig Gleeson, Sergey Khayrulin

**Affiliations:** 1 Department of Physiology, Development and Neuroscience, University of Cambridge, Cambridge, CB2 3DY, UK; 2 Lab. of complex systems simulations, A.P. Ershov Institute of Informatics Systems, Siberian Branch of the Russian Academy of Sciences, Novosibirsk, 630090, Russian Federation; 3 Department of Neuroscience, Physiology and Pharmacology, University College London, London, UK

## 

OpenWorm is an international collaboration with the aim of producing an integrative computational model of Caenorhabditis elegans to further the understanding of how macroscopic behaviour of the organism emerges from aggregated biophysical processes. A core component of the project involves the integration of electrophysiological modelling and predictive-corrective incompressible smoothed particle hydrodynamics (PCISPH) to model how neuronal and muscle dynamics effect the nematode's behaviour. Several tools are being utilised and developed in the course of the project:

• Electrophysiological model parameters are constrained to reproduce experimental measurements using the Optimal Neuron toolkit [1]

• A PCISPH solver is under development [2] - a combination of general PCISPH algorithms proposed by [3], boundary-handling algorithms proposed by [4], a surface tension model based on [5] and our own implementation of elastic matter and biophysics-specific features, as well as parallelization, optimization and tuning. It is the first open source, parallel OpenCL/C++ PCISPH high-performance implementation.

• A generic model integration framework (Gepetto [6]) will be used to integrate electrophysiology and body-wall interactions

• All electrophysiological models are NeuroML-compatible [7].

• The Open Worm Browser provides a powerful way to visualise C. Elegans anatomy [8]

All of the above mentioned applications are open source, freely available and can be used for modelling other neuronal systems.
